# Health related quality of life and psychological parameters in different polycystic ovary syndrome phenotypes: a comparative cross-sectional study

**DOI:** 10.1186/s13048-021-00811-2

**Published:** 2021-04-24

**Authors:** Bahadori Fatemeh, Jahanian Sadatmahalleh Shahideh, Mirzaei Negin

**Affiliations:** Department of Reproductive Health and Midwifery, Faculty of Medical Sciences, Tarbiat Modares University, Jalal Al-Ahmad Highway, Nasr Bridge, Tehran, Iran

**Keywords:** Polycystic ovary syndrome, Phenotypes, Quality of life, Anxiety, Depression

## Abstract

**Background:**

Polycystic Ovary Syndrome (PCOS) is associated with high levels of phsychological implications and detriments to Quality of Life (QoL). The aim of this study was to assess Health- Related Quality of Life (HRQoL), depression, and anxiety in Iranian women with different PCOS phenotypes.

**Methods:**

The present observational, cross-sectional study was carried out on 239 PCOS women who were classified on the basis of Rotterdam criteria into four categories: A (*n* = 77), B (*n* = 38), C (*n* = 68), and D (*n* = 56). They asked to fill out three questionnaires, namely, HRQoL, SF-12, and HADS.

**Results:**

No significant differences were observed between the four PCOS phenotypes for anxiety, depression and QoL, as well as HRQoL domains related to infertiliy, weight and emotional problems (*P* > 0.05). Phenotypes A and B had worse HRQoL related to hirsutism (13.98 ± 5.22, 14.13 ± 6.23, *P* < 0.001). In addition, no significant differences were observed between them for HRQoL domains. While the score of acne in phenotype D (19.60 ± 5.12, *P* = 0.003) and menstrual score in phenotype C were significantly higher comparing to the other PCOS groups (16.82 ± 3.87, *P* < 0.001).

**Conclusion:**

Presenting similar psychological profiles in all phenotypes unveils the importance of pychological well-being screening, even in milder reproductive phenotypes.

## Introduction

Polycystic Ovary Syndrome (PCOS) is a frequent endocrine disorder that affects 7.1% of Iranian reproductive-aged women [1]. It is a heterogeneous condition that possesses a remarkably broad spectrum of clinical symptoms and affects many organs systems [2]. Additionally, PCOS is accompanied by multiple painful and unfavorable symptoms such as infertility, irregular menstruation, acne, obesity, scalp hair thinning, and excess hair growth, which culturally have been defined as unpleasant and unfeminine features and have adverse effects on psychological well-being in women [3]. Hence, these are manifested when finding a partner and sexual activity are important. The cosmetic and psychosexual implications of PCOS may lead to serious emotional distress in affected women [4]. This array of undesirable symptoms not only can have a profound impact on psychological morbidity, but the ranges of treatments that are offered to relieve them also are a vast contributor to an overall diminished woman’s quality of life (QoL) [5].

PCOS is diagnosed based on Rotterdam criteria, two of three characteristics: ultrasound Polycystic Ovarian Morphology (PCOM), Hyperandrogenism (HA), and chronic Ovulatory Dysfunction (OD) [6]. Four different phenotypes of PCOS have been introduced by using a possible combination of these criteria: phenotype A includes HA + OD + PCOM, phenotype B and C comprise HA + OD and HA + PCOM, respectively, and phenotype D contains OD+ PCOM [7]. Although, it seems that these different phenotypes of PCOS differ in terms of hormonal, anthropometric, and metabolic indices [8], there are limited researches to assess reproductive features and metabolic profiles of these diagnostic categories [9]. Therefore, it is not completely obvious that these phenotypes suffer from the same negative psychological health risks or not. In addition, to our knowledge there is currently no research comparing psychological aspects of different PCOS phenotypes in Iranian papulation.

Given the crucial role of psychological profile in the chronic diseases like PCOS and their impact on self-management, the ability to enhance lifestyle, adherence to therapy, and pursue treatment [10], assessing their psychological status seems to be necessary. The aim of this study was to compare the psychological features such as anxiety, depression, and HRQoL in women with different PCOS phenotypes.

## Materials and methods

In this cross-sectional study, first, a pilot study was conducted on 22 women based on the deviation of QoL score (s). Then, using the appropriate formula at 95% confidence interval and error 3 (d), it was found that a sample size of 215 women was needed with 10% sample loss:

Sample size estimation formula: $n=\frac{\left({z}_{1-\frac{\alpha }{2}}\right){s}^2}{d^2}$ $n=\frac{\left({z}_{1-\frac{\alpha }{2}}\right){s}^2}{d^{2.}}$

Overall, 239 sexually active women (aged 18–45 years), who had PCOS (77 with phenotype A, 38 with phenotype B, 68 from phenotype C, and 56 from phenotype D), and reffered to a gynocology clinic of Arash Hospital, Tehran Province (Iran) whitin 2018–2019 were included in this study through convenience sampling method.

The inclusion criteria were: women of reproductive age (18–45 years), married and non-pregnant, having PCOS based on Rotterdom criteria and willing to participate in the study. Also to avoid possible confounding factors, the exclusion criteria included breastfeeding, suffering from chronic diseases (such as diabetes, hypertension,..), endocrinological abnormalities (like primary hyperprolactinemia, thyroid dysfunction, Cushings’ syndrome, congenital adrenal hyperplasia, and androgen producing neoplasm), drug and alcohol addiction, taking any antidepressant drugs, and those on hormonal medications for 3 months prior to the study, including oral contraceptives, glucocorticoids, ovulation induction agents, and estrogenic or anti-androgenic medications, which might influence on the serum androgen levels.

The participants were asked to complete three self-report questionnaires that had been tested for validity and reliability. They inclouded Hospital Anxiety and Depression Scale (HADS), Short Form health survey (SF-12), and the Modified Polycystic Ovary Syndrome Health-Related Quality of Life Questionnaire (MPCOSQ).

This study was approved by the Ethics Committee of Tarbiat Modares University (IR.MODARES.REC.1397.153). In addition, all patients participated voluntarily, and signed informed consent.

### Phenotypical features

Anthropometric measurements including body weight, height, Hip Circumference (HC), and Waist Circumference (WC) were performed by a single technician for all participants. Body weight (kg) and height (cm) were measured by erect posture and without shoes. Body Mass Index (BMI) calculated by dividing the weight (kg) by height (m) square to assess the obesity [11]. WC (cm) was measured between the costal margin and the pelvic brim, and HC at the level of the symphysis pubis. Dividing WC by the HC was considered as the Waist to Hip Ratio (WHR).

### Clinical features

All clinical findings including hirsutism, acanthosis nigricans, galactorrhea, and menstural cycle characteristics were evaluated by a gynechologist. Oligo/anovulation was defined as delaying of menses > 35 days or < 8 spontaneous hemorrhagic episodes in a year. Clinical hyperandrogenism was measured by Modified Ferriman Gallwey (m-FG), which was used for scoring the presence of terminal hairs over nine body areas (i.e. upper lip, chin, chest, upper and lower abdomen, thighs, upper and lower back, and upper arms) from 0 to 4, and m-FG score ≥ 8 was considered hirsutism [12].

### Para-clinical assessment

In the early follicular phase (days 3–5) of spontaneous bleeding or withdrawal bleeding induced by medroxyprogesterone acetate, laboratory assessments and ultrasound sonographies were performed.

All participants underwent abdominal ultrasonography by a sonologist; ovaries containing 12 or more follicles measuring 2–9 mm in diameter and/or enlarged ovarian volume (> 10 mm^3^) were considered as a positive polycystic sonographic morphology [6].

All blood samples’ test was done after 8-h fasting and overnight bed rest in Arash Hospital laboratory. The laboratory investigation consisted of Sex Hormone-Binding Globulin (SHBG), Total Testosterone (TT) levels, and Free Androgen Index (FAI) which were computed by TT (nmol/L)/SHBG (nmol/L) × 100). Hyper androgenism was defined as TT greater than 0.68 ng/ml was greater than 5.36%. All of the above tests were assessed by commercial kits (Pars Azmoon Inc., Tehran, Iran) using Auto-analyzer BT2000 device. Biochemical measurement of TT and SHBG levels was carried out using Cobas E411 device based on electro-chemiluminescence method (Roche Instr Kit, Germany).

### Questionnaire

The participants were asked to complete the folloeing self-report questionnaires:

Demographic survey that included the age of the participants, the number of children, their educational levels, occupational status, type of previous deliveries infertility status, and medical history.

### Health-related quality of life (HRQoL)

The Modified Polycystic Ovary Syndrome Health-Related Quality of Life Questionnaire (MPCOSQ) is a valid measure of HRQoL in PCOS patients. It includes 30 seven-point Likert scale questions from six HRQoL areas or domains: emotional disturbances (8 items), hirsutism (5 items), infertility (4 items), weight (5 items), menstrual (4 items) and acne (4 items). The total score of each field is based on the score obtained divided by the number of questions asked in that field. In sum, the largest HRQoL impairment in each field is indicated by a score of 1, and 7 sugesstes no problems or difficulties [13]. The validity and reliability of this questionnaire have been confirmed in Iran [14].

### Quality of life (QoL)

QoL was measured by a Short Form Health Survey (SF-12), which contains a total of eight ereas, namely physical functioning, physical role, bodily pain, general health, vitality, social functioning, role emotional, and mental health by 12 questions. Higher scores indicate better QoL [15]. The validity and reliability of the Iranian version of this questionnaire have been confirmed [16].

### Depression and anxiety

The Hospital Anxiety and Depression Scale (HADS) objectively assesses depression and anxiety. This instrument consists of 14 questions (seven items in each subscal) with the score range of 0–3. In sum, higher scores represent higher symptom levels for both subscales, and scores equal to or above 11 are considered a clinical disease [17]. The validity and reliability of the Iranian version of HADS have been confirmed [18].

### Statistical analysis

Quantitative variables were presented as Mean ± Standard Deviation (M ± SD); however, qualitative variables were reported as proportions. First of all, the quantitative variables were assessed for normality using the Kolmogorov–Smirnov’s (KS) test. One-way ANOVA and Kruskal-Wallis (KW) tests were applied for normal and non-normal/ordinal variables, respectively. The Mann-Whitney’s U test (MW) was performed for pairwise comparison of the group if there was a significant group effect. Then Bonferroni’s correction performance (*P* < 0.008) was considered significant. The MW’s test was used for compareing the normal variables between two PCOS subgroups and qualitative variables were compared by the Chi-square test. Statistical significance was set at *P* < 0.05. Data were analyzed by the SPSS software (Statistical Package for the Social Sciences, version 22.0, SPSS Inc., Chicago, IL, USA).

## Results

Table 1 gives some information about the basic features of PCOS diagnosis in each group at the beginning of the study.

**Table 1:** Basic features of PCOS diagnosis in each group

Variable	HA + PCOM + OD (A) ***n*** = 77	HA + OD (B) ***n*** = 38	HA + PCOM (C) ***n*** = 68	OD + PCOM (D) ***n*** = 56
**TT (nmol/L**	2.58 ± 1.74	1.81 ± 0.35	1.60 ± 0.79	1.10 ± 0.28
**FAI**	6.14 ± 4.02	4.64 ± 1.03	3.16 ± 1.59	2.23 ± 0.66
**SHBG (nmol/L)**	41.48 ± 4.73	39.67 ± 5.56	51.06 ± 3.17	50.58 ± 9.66
**FG score**	9.29 ± 2.01	8.71 ± 1.51	9.09 ± 1.49	2.49 ± 2.19
**Ovarian follicle counts**	14.41 ± 0.90	5.70 ± 3.09	14.61 ± 1.02	13.90 ± 1.89

Data are given as Mean ± SD. *TT* Total Testosterone. *FAI* Free Androgen Index. *SHBG* Sex Hormone Binding Globulin. *FG-score* Ferriman–Gallwey score

Table 2 shows an overview of the demographic characteristics of women with different phenotypes of PCOS. The age of the participants ranged from 20 to 43 years. As can be seen, no significant difference was observed between the PCOS subgroups in terms of age, BMI, educational level, occupation, WHR, and infertility status (*P* > 0.05).

**Table 2:** Comparison of demographic characteristics between different PCOS phenotypes

Valuable	HA + PCOM + OD (A) ***n*** = 77	HA + OD (B) ***n*** = 38	HA + PCOM (C) ***n*** = 68	OD + PCOM (D) ***n*** = 56	***P***-value
**Age (years)**^**a**^	29.18 + 5.71	31.55 + 5.68	31.67 + 5.05	31.28 + 5.5	0.26
**BMI (Kg/m**^**2**^**)**^a^	27.21 + 4.80	26.59 + 3.85	25.60 + 3.67	26.45 + 3.75	0.14
**Number of children**					0.05
**0**	63 (81.8)	20 (52.6)	48 (70.6)	40 (71.4)	
**1**	13 (16.9)	15 (39.5)	17 (25)	14 (25)	
**2**	1 (1.3)	2 (5.3)	3 (4.4)	2 (3.6)	
**3**	0 (0)	1 (2.6)	0 (0)	0 (0)	
**Number of abortion**					0.007
**0**	68 (88.3)	29 (76.3)	47 (69.1)	47 (83.9)	
**1**	5 (6.5)	5 (13.2)	13 (19.1)	7 (12.5)	
**2**	4 (5.2)	4 (10.5)	6 (8.8)	0 (0)	
**3**	0 (0)	0 (0)	2 (2.9)	2 (3.6)	
**WHR**^a^	0.87 ± 0.021	0.88 ± 0.023	0.87 ± 0.026	0.87 ± 0.016	0.77
**Occupation status**^b^					0.50
**House Wife**	71 (92.9)	35 (92.1)	58 (85.29)	50 (89.28)	
**Employed**	6 (7.79)	3 (7.89)	10 (14.7)	6 (10.71)	
**Educational level**^b^					0.49
**Less than a diploma**	24 (31.16)	11 (28.94)	10 (14.7)	19 (33.9)	
**Diploma**	31 (40.25)	14 (36.84)	26 (38.23)	17 (30.35)	
**Higher than diploma**	22 (28.59)	13 (34.22)	32 (47.07)	20 (35.75)	
**Infertility status**^b^					0.56
**Yes**	66 (85.71)	28 (73.68)	58 (85.29)	48 (85.71)	
**No**	11 (14.28)	10 (26.31)	10 (14.7)	8 (14.28)	

*HA* Hyperandrogenism, *OD* Ovulatory Dysfunction, *PCOM*:Polycystic Ovarian Morphology, *BMI*: Body Mass Index. *WHR* Waist Hip Circumference Ratio, *PCOS* Polycystic Ovary Syndrome

^a^ Values are given as mean ± SD by using Kruskal-Wallis test

^b^ Values are given as a number (%) by using Chi-square test

### Health-related quality of life

Table 3 presents the summery statistics for MPCOSQ in different phenotypes of PCOS. It can be seen that there are no statistically significant differences between the different phenotype categories in the scores of infertility, weight, and emotional disturbance (*P* > 0.05). Additionally, there are no significant differences between phenotypes A and B in all domains. Although phenotypes A and B indicate lower scores in the hirsutism subgroup (13.98 ± 5.22, 14.13 ± 6.23 respectively), phenotype D with 23.33 ± 4.81 expresses higher score in this subgroup. Also, in the acne and menstrual subgroups phenotype D and C reveal higher scores respectively (*P* < 0.001).

**Table 3:** Comparison of MPCOSQ scores between different PCOS phenotypes

Valuable	HA + PCOM + OD (A) ***n*** = 77	HA + OD (B) ***n*** = 38	HA + PCOM (C) ***n*** = 68	OD + PCOM (D) ***n*** = 56	***P***-value*	pair wise comparison; ***P***-value**
**Hirsutism**	13.98 ± 5.22	14.13 ± 6.23	17.22 ± 5.28	23.33 ± 4.81	< 0.001	A &B: 0.75 A&D:< 0.001 A&C:< 0.001 B&D:< 0.001 B&C: 0.002 D&C: 0.001
**Acne**	17.28 ± 5.39	16.15 ± 5.49	16.73 ± 4.99	19.60 ± 5.12	0.003	A &B: 0.34 A&D: 0.007 A&C: 0.498 B&D: 0.004 B&C: 0.668 D&C:< 0.001
**Menstrual**	12.29 ± 3.91	12.28 ± 3.96	16.82 ± 3.870	13.89 ± 4.36	< 0.001	A&B: 0.613 A&D: 0.234 A&C:< 0.001 B&D: 0.48 B&C:< 0.001 D&C:< 0.001
**Weight**	17.90 ± 6.80	19.23 ± 7.29	20.45 ± 7.24	19.42 ± 8.08	0.15	–
**Infertility**	9.85 ± 4.71	11.26 ± 7.44	10.75 ± 5.95	9.75 ± 5.77	0.723	–
**Emotional**	19.93 ± 7.16	20.36 ± 10.02	20.95 ± 7.76	19.94 ± 8.79	0.762	
**Total**	91.27 ± 20.87	93.5 ± 26.53	102.94 ± 23.11	105.32 ± 26.28	0.002	A&B: 0.91 A&D: 0.003 A&C: 0.002 B&D: 0.021 B&C: 0.029 D&C: 0.77

*HA* Hyperandrogenism, *OD* Ovulatory Dysfunction, *PCOM* Polycystic Ovarian Morphology, *PCOS* Polycystic Ovary Syndrome

MPCOSQ; Modified Polycystic Ovary Syndrome Health-Related Quality of Life

* *P*-values refer to the Kruskal-Wallis test

** Kruskal-Wallis test followed by appropriate post hoc test (Mann-Whitney U) (Bonferroni correction was used for multiple comparisons, *P* < 0.008 was considered significant)

### Depression and anxiety

Table 4 compares the mean scores of different domains of HADS in different phenotypes of PCOS. There are no statistically significant differences between the mean scores of depression, anxiety, and total HADS between the different phenotype categories (*P* > 0.05).

**Table 4:** Comparing the scores of HADS between different PCOS phenotypes

Variable	HA + PCOM + OD (A) ***n*** = 77	HA + OD (B) ***n*** = 38	HA + PCOM (C) ***n*** = 68	OD + PCOM (D) ***n*** = 56		***P***-value*
**Anxiety**	10.74 ± 4.21	11.73 ± 3.39	10.50 ± 3.61	9.82 ± 4.19		0.245
**Depression**	6.74 ± 3.73	8.43 ± 3.91	6.37 ± 3.78	6.29 ± 3.50		0.092
**Total**	17.49 ± 7.32	20.16 ± 6.82	16.87 ± 6.80	16.11 ± 6.8		0.101

*HA* Hyperandrogenism, *OD* Ovulatory Dysfunction, *PCOM* Polycystic Ovarian Morphology, *PCOS* Polycystic Ovary Syndrome, *HADS* Hospital Anxiety and Depression Scale

* *P*-values are given as Mean ± SD by using Kruskal-Wallis test

### Quality of life

Table 5 provieds the results obtained from summerizing the mean scores of QoL domains in the PCOS phenotype categories. As shown, there are no ststistically significant differences betwee the different phenotype categories (*P* > 0.05).

**Table 5:** Comparison of SF-12 mean scores between different PCOS phenotypes

Variable	HA + PCOM + OD (A) ***n*** = 77	HA + OD (B) ***n*** = 38	HA + PCOM (C) ***n*** = 68	OD + PCOM (D) ***n*** = 56	***P***-value
PF^a^	60.90 ± 31.46	64.16 ± 26.81	66.51 ± 30.25	71.56 ± 28.29	0.338
RP^a^	55.68 ± 21.89	54.16 ± 22.58	54.24 ± 23.02	64.21 ± 23.85	0**.**101
BP^a^	66.81 ± 24.55	70.83 ± 28.68	64.28 ± 25.16	69.60 ± 25.15	0.491
GH^a^	50.45 ± 26.12	36.66 ± 14.28	48.66 ± 21.00	45.58 ± 19.81	0.076
VT^a^	54.09 ± 24.42	55.00 ± 24.91	55.80 ± 24.76	52.94 ± 27.67	0.914
SF^a^	61.81 ± 28.40	51.66 ± 24.50	58.03 ± 22.91	57.35 ± 25.63	0.242
RE^a^	50.45 ± 22.68	45.00 ± 23.80	55.58 ± 23.22	57.10 ± 26.36	0.163
MH^a^	60.68 ± 22.62	59.58 ± 16.30	57.36 ± 18.73	59.80 ± 19.25	0.865
MCS^b^	41.70 ± 10.40	39.11 ± 8.83	41.40 ± 9.66	40.89 ± 10.18	0.692
PCS^b^	43.72 ± 7.75	43.66 ± 6.50	43.77 ± 7.52	45.73 ± 7.40	0.434
Total.SF-12^a^	546.33 ± 139.22	519.8 ± 6104.62	545.67 ± 128.23	564.81 ± 136 + 89	0.558

*HA* Hyperandrogenism, *OD* Ovulatory Dysfunction, *PCOM* Polycystic Ovarian Morphology, *PCOS* Polycystic Ovary Syndrome, *SF-12* The 12-item Short Form Health Survey, *PF* Physical Functioning, *RP* Role Physical, *BP* Bodily Pain, *GH* General Health, *VT* Vitality, *SF* Social Functioning, *RE* Role Emotional, *MH* Mental Health, *PCS* Physical Component Summary; *MCS* Mental Component Summary

^a^ Values are given as M ± SD by using Kruskal-Wallis test

^b^ Values are given as M ± SD by using One-Way ANOVA

Table 6 shows the correlation between the number of abortion and depression, anxiety and QoL, and there are no any association between them (*P* > 0.05).

**Table 6:** Correlation between the number of abortion and anxiety, depression, and MPCOSQ

Variables	R	***P***-value*	Mean ± SD
**Anxiety**	−0.009	0.899	10.58 ± 3.93
**Depression**	0.034	0.64	6.78 ± 3.76
**Total HADS**	0.013	0.858	17.36 ± 7.03
**Total MPCOSQ**	−0.074	0.30	546.91 ± 130.32

*HADS* Hospital Anxiety and Depression Scale, *MPCOSQ* Modified Polycystic ovary Syndrome, *HRQoQ* Health-Related Quality of life Questionnaire

* *P*-values and r are given by Pearson’s correlation

As can be seen from Table 7, the average scores of emotion and QoL were lower in the infertile group. Also the women in this group feel more an anxiety.

**Table 7:** Comparison of variable scores in fertile and infertile groups

Variables	Infertility	N	Mean	***P***-value
**Total FSFI**	no	39	25.04 ± 3.60	0.57
	yes	200	24.58 ± 4.12	
**Hirsutism**	no	39	17.20 ± 6.57	0.96
	yes	200	17.14 ± 6.52	
**Infertility**	no	39	15.69 ± 7.20	0.001
	yes	200	9.21 ± 4.76	
**Weight**	no	39	18.71 ± 7.31	0.62
	yes	200	19.36 ± 7.32	
**Mensuration**	no	39	14.97 ± 5	0.07
	yes	200	13.57 ± 4.33	
**Acne**	no	39	18.76 ± 5.12	0.10
	yes	200	17.24 ± 5.36	
**Emotional Disturbance**	no	39	23.74 ± 8.53	
	yes	200	19.62 ± 7.97	0.004
**Total MPCOSQ**	no	39	109.13 ± 25.82	0.002
	yes	200	96.16 ± 23.74	
**Depression**	no	39	5.48 ± 3.50	0.04
	yes	200	7.01 ± 3.76	
**Anxiety**	no	39	10.10 ± 3.72	0.48
	yes	200	10.66 ± 3.98	
**Total HADS**	no	39	15.58 ± 6.52	0.14
	yes	200	17.68 ± 7.09	
**Total BICI**	no	39	43.48 ± 14.28	0.44
	yes	200	41.05 ± 15.90	
**Self-steam**	no	39	12.65 ± 3.44	0.30
	yes	200	11.94 ± 3.35	
**Total SF.12**	no	39	5.5282E2	0.79
	yes	200	5.4587E2	

*MPCOSQ* Modified Polycystic Ovary Syndrome, Health-Related Quality of Life. *HADS* Hospital Anxiety and Depression Scale. *SF-12* The 12-item Short Form Health Survey. *FSFI* Female Sexual Function Index

## Discussion

PCOS is one of the most common endocrine disorders in women with reproductive age. The adverse impact of diverse clinical manifestations and prolonged complication on worsened QoL and psychological impairments have become a new area of research in the last decades [19]. Although it is well-established that PCOS women suffer from anxiety, depression, and markedly reduced QoL [5, 20], the association between psychological disorder and each phenotypical category has remained unclear. Therefore, this study was conducted to compare emotional well-being, anxiety and depression among different PCOS phenotypes in Iranian women.

### “Classic” PCOS (phenotypes a and B)

“Classic “PCOS, which includes phenotypes A and B, is classified based on chronic OD and HA. Also finding PCOM with sonography leads to distinction between these two phenotypes [7]. In the current study, comparing different dimensions of MPCOSQ across confirmed phenotypic categories of PCOS revealed that classic PCOS represents sever hirsutism symptoms. Also there is not any significant difference between phenotypes A and B.

It is important to note that hirsutism is defined as the excessive terminal hair growth in androgen-dependent areas with male distribution of women body [21]. Not only may hirsutism be an unequivocal marker of excessive androgen action [22], it is also influenced by the peripheral metabolism of androgens, insulin resistance and compensatory hyperinsulinism [23]. A possible explanation for these results might be the similarity of classic PCOS presentation. Indeed, classic PCOS is accompanied by abdominal obesity, increased levels of LH and LH/FSH ratio, increased level of androgens, and elevated insulin and insulin resistance [24].

The soaring total score of MPCOSQ indicates perturbed HRQoL in classical PCOS patients. These results seem to be consistent with the findings of other researchers who found impaired HRQoL functioning in depressed compared with non-depressed women with PCOS [25]. Although hair growth and menstrual disturbance were reported as the most distressing factors in a Turkish investigation [25], Greenwood et al. [26], suggested infertility and weight problems as the most serious concerns. This inconsistency may be due to clinical compositions and perhaps cultural differences.

### “Ovulatory PCOS” (phenotype C)

Patients with “ovulatory PCOS”, generally, seem to have the mild form of classic PCOS. Indeed, phenotype C PCOS patients manifest average values (compared with patients with other phenotypes of PCOS) of serum androgens, insulin, and testosterone, atherogenic lipids, hirsutism scores, and prevalence of metabolic syndrome [7]. Also Guastella et al. [24] reported intermediate levels of BMI, waist circumference, and testosterone in ovulatory PCOS patients compared with classic PCOS patients and control group. Additionally, normal levels of LH have been described in these patients as the main difference with other phenotypes [24]. One of the issues that emerges from these findings is that passaging from classic phenotypes to ovulatory PCOS and vice versa can be related to socio-cultural and environmental effects [27].

The data collected from 1297 Greek women with PCOS demonstrated that menstrual cycle pattern is more irregular in the “classic” PCOS phenotypes as compared with phenotype D; however, its appear to normalize with ageing and independent of obesity [28].

One unanticipated finding was that phenotype C patients reported lower menstrual abnormalities in this study. Also previous investigations revealed discrepant results regarding the relationship between obesity and menstrual cycle abnormalities. Some prior studies have noted that there are not any differences between irregular menstruation and obesity [28, 29]; however, some others identified higher rate of menstrual irregularity in overweight women [30].

### “Non-hyperandrogenic PCOS” (phenotype D)

Non-hyperandrogenic PCOS patients have the mildest degree of endocrine and metabolic dysfunction as compared with healthy controls. They have lower LH to FSH ratios, lower total and free T levels, and higher sex hormone-binding globulin levels as compared with classic PCOS patients [7].

In the current study, the mean score of acne in phenotype D was higher rather than in other phenotypes. This result can be explained by the well-established fact the presence of acne has been related to clinical and/or biochemical HA [31]. MJ Chen et al. [32] also demonstrated that presence of acne was related with high level of serum dehydroepiandrosterone sulfate (DHEAS) in PCOS women. It is worth to mention that in all cases of this study, despite the lack of significant differences between the groups in terms of BMI, WHR, and hormones levels, phenotype D was associated with lower acne symptoms.

One striking observation in the present work was that the HRQoL in classic phenotype is more impaired as lower total scores. However, there is an inconsistency in identifying the most important contributing factor to impaired HRQoL. For instance, Moridi et al. [33] argued that the average of HRQoL and all the six subscales of MPCOSQ in the PCOS women were significantly lower than in the healthy group. Similarly, Bazarganipour et al. [34] considered the negative impact of PCOS on HRQoL, although it was not obvious that impairment domains had the most adverse effect and the relative degree of impairment varied among the different samples. Nevertheless, in their meta-analysis [35] menstrual irregularities and infertility were reported as the most affected domains in worsened HRQoL than the obesity. Likewise, Yoldemir et al. [36] hold the view that menstrual problems were the worst parameter affecting the QoL.

The findings of this study supports evidence from a previous research that evaluated clinical features and HRQoL between infertile women with PCOS phenotypes and women with unexplained infertility [37]. The association of Ferriman Gallwey’s scores, phenotype classic PCOS, and primary infertility with lower HRQoL were identified by Dilbaz et al. [37]. Also, Moran et al. [38], who compared different PCOS phenotypes based on the National Institute of Health (NIH) criteria (HA and OD), reported poorer HRQoL in women with NIH PCOS compared with non-NIH PCOS. It seems possible that this result is due to the significant effects of clinical HA which (such as excess body hair) on the perception of women as feminine. Hence, psychological well-being was reported to be more related to hirsutism in South Asians rather than in white Europeans with PCOS [39].

### Quality of life, depression and anxiety

Several studies have confirmed the effectiveness of PCOS on psychological features like soaring anxiety and depression [40], and worsening of QoL [41]. This study like to obtain data that help to address different psychological manifestations of PCOS phenotypes. To assess the psychological features, Moran et al. [38] compared different PCOS phenotypes based on the National Institute of Health (NIH) criteria (HA and OD(. They reported similar anxiety and depression levels in woman with NIH and non-NIH PCOS. Consistent with the literature, the present research found no significant differences between depression, anxiety and various dimensions of QoL in different PCOS phenotypes. This emphasizes the intricacy of psychological dysfunction in PCOS. Although, it is difficult to explain this result, it might be related to the fact that PCOS challenges their feminine identity encompassing infertility, acne, hirsutism, and obesity. Consequently, it is highly likely that all these symptoms can possess detrimental effects on QoL and mental health. Moreover, They potentially precipitate depression and anxiety [42].

The present study is one of the first attempts to thoroughly perceive differences between PCOS phenotypes in terms of QoL, HRQoL, depression, and anxiety. Furthermore, it provided an important opportunity to advance the understanding of the requirement of specific mental health screening for each phenotype. Indeed, lifestyle modification as the first- line of beneficial management depends on self-efficacy and psychological well-being. Despite the appropriate study population (contrary to the previous studies), this study was limited by the absence of complete information about family history and previous mental illnesses. Further research is required estimate female sexual dysfunction in each phenotype’s categories and establish the similarities of psychological parameters.

## Conclusion

In this study, it was observed that although presenting similar psychological profiles and QoL scores, phenotype categories reveal decrement in different HRQoL domains.

## Data Availability

The data sets used and analyzed during the current study are available from the corresponding author on reasonable request.

## Abbreviations

MPCOSQ: Modified polycystic ovary syndrome health-related quality of life questionnaire
HADS: Hospital anxiety and depression scale
SHBG: Sex hormone-binding globulin
PCOM: Polycystic ovarian morphology
HRQOL: Health-related quality of life
DHEAS: Dehydroepiandrosterone sulfate
M-FG: Modified ferriman gallwey
PCOS: Polycystic ovary syndrome
SF-12: Short form health survey
NIH: National institute of health
OD: Ovulatory dysfunction
FAI: Free androgen index
WC: Waist circumference
BMI: Body mass index
WHR: Waist to hip ratio
HA: Hyperandrogenism
TT: Total testosterone
HC: Hip circumference
MW: Mann-whitney
Qol: Quality of life
